# Association of 3-Phenoxybenzoic Acid Exposure during Pregnancy with Maternal Outcomes and Newborn Anthropometric Measures: Results from the IoMum Cohort Study

**DOI:** 10.3390/toxics11020125

**Published:** 2023-01-27

**Authors:** Juliana Guimarães, Isabella Bracchi, Cátia Pinheiro, Nara Xavier Moreira, Cláudia Matta Coelho, Diogo Pestana, Maria do Carmo Prucha, Cristina Martins, Valentina F. Domingues, Cristina Delerue-Matos, Cláudia C. Dias, Luís Filipe R. Azevedo, Conceição Calhau, João Costa Leite, Carla Ramalho, Elisa Keating, Virgínia Cruz Fernandes

**Affiliations:** 1 CINTESIS@RISE, Department of Biomedicine, Unit of Biochemistry, Faculty of Medicine, University of Porto, 4200-319 Porto, Portugal; 2 CINTESIS@RISE, Faculty of Medicine, University of Porto, 4200-319 Porto, Portugal; 3 Department of Nutrition and Dietetics (MND), Faculty of Nutrition Emília de Jesus Ferreiro (FNEJF), Fluminense Federal University (UFF), Niterói 20010-010, RJ, Brazil; 4 CINTESIS@RISE, Nutrition and Metabolism, NOVA Medical School│FCM, Universidade Nova de Lisboa, 1169-056 Lisboa, Portugal; 5 Department of Obstetrics, Centro Hospitalar Universitário S. João, 4200-319 Porto, Portugal; 6 REQUIMTE/LAQV, Instituto Superior de Engenharia, Politécnico do Porto, 4249-015 Porto, Portugal; 7 CINTESIS@RISE, Department of Community Medicine, Information and Health Decision Sciences (MEDCIDS), Faculty of Medicine, University of Porto, 4200-319 Porto, Portugal; 8 Department of Ginecology-Obstetrics and Pediatrics, Faculty of Medicine, University of Porto, 4200-319 Porto, Portugal; 9 Instituto de Investigação e Inovação em Saúde, i3S, Universidade do Porto, 4200-135 Porto, Portugal

**Keywords:** 3-PBA, pyrethroid pesticides, pregnancy, newborn, anthropometry

## Abstract

The aims of this study were to characterize the exposure of pregnant women living in Portugal to 3-phenoxybenzoic acid (3-PBA) and to evaluate the association of this exposure with maternal outcomes and newborn anthropometric measures. We also aimed to compare exposure in summer with exposure in winter. Pregnant women attending ultrasound scans from April 2018 to April 2019 at a central hospital in Porto, Portugal, were invited to participate. Inclusion criteria were: gestational week between 10 and 13, confirmed fetal vitality, and a signature of informed consent. 3-PBA was measured in spot urine samples by gas chromatography with mass spectrometry (GC-MS). The median 3-PBA concentration was 0.263 (0.167; 0.458) µg/g creatinine (*n* = 145). 3-PBA excretion was negatively associated with maternal pre-pregnancy body mass index (BMI) (*p* = 0.049), and it was higher during the summer when compared to winter (*p* < 0.001). The frequency of fish or yogurt consumption was associated positively with 3-PBA excretion, particularly during the winter (*p* = 0.002 and *p* = 0.015, respectively), when environmental exposure is low. Moreover, 3-PBA was associated with levothyroxine use (*p* = 0.01), a proxy for hypothyroidism, which could be due to a putative 3-PBA—thyroid hormone antagonistic effect. 3-PBA levels were not associated with the anthropometric measures of the newborn. In conclusion, pregnant women living in Portugal are exposed to 3-PBA, particularly during summer, and this exposure may be associated with maternal clinical features.

## 1. Introduction

Pesticides belong to a large family of compounds used to control insect (insecticides, insect repellents), weeds (herbicides), microbe (fungicides, disinfectants), or mouse and rat (rodenticides) pests [1].

Synthetic pyrethroids (SPs) belong to the class of insecticides and are commonly used. These insecticides derive from natural compounds produced by some species of chrysanthemum flowers, the so-called pyrethrins, and they are used to manage pests both in agriculture and in residences and to reduce the transmission of diseases acquired by insects [2,3].

3-phenoxybenzoic acid (3-PBA) is a general metabolite of several SPs, which is used as a biomarker of SP exposure [4].

In northern Portugal, three studies were carried out to characterize agricultural soil sample contamination with 3-PBA. Those studies showed that 3-PBA was detectable at the range of ng per g of soil, in samples of the north of Portugal [2,5,6]. One other study showed that pyrethroids interfere with the germination and development of plants since their phototoxicity can alter the levels of chlorophyll and carotenoids [4]. The presence of pyrethroids in agricultural soils indicates the relevance of extending monitoring programs for the analysis of these compounds in soils and soil-borne foods [2,5,6].

For example, 75% of fruits and vegetable samples labelled as ‘organic’ (including tomatoes, oranges, grapes, apples, bananas, onions, lettuce, green peppers, carrots, and broccoli) collected from grocery stores in North Carolina, USA, had measurable levels of at least one pyrethroid [7].

The 2018 European Union (EU) report on pesticide residues in food showed that Portugal was one of the countries with the highest maximum residue levels (MRLs) exceeding rates, but none of the residues detected to exceed MRLs were pyrethroids [8].The overall results of this report suggest that the levels of pesticides assessed in the food products analyzed are unlikely to pose a concern for the health of the consumer [8].

Besides food, pyrethroids are also found in pet shampoos, medication used for treating scabies, and topical louse treatments [9].

Exposure to pesticides in the population is widespread, especially via dermal, ingestion, and inhalation routes, and consequently, SPs may enter the food chain, affecting the environment and human health [10].

3-PBA found in the human body may result from absorption of 3-PBA resulting from environmental degradation of several SPs [2], or it may result from the endogenous hydrolysis of SPs by mammalian carboxylesterases (CEs) [11].

Once in the human body, pyrethroid compounds, including 3-PBA, are known to be transported by the placenta, since detectable levels of permethrin were found in cord blood samples collected upon delivery [12].

The developing nervous system is highly susceptible to the neurotoxicity of pesticides, as well as to many types of environmental toxicants. This heightened sensitivity occurs not only during prenatal but also postnatal development, extending into adolescence [13]. Impacts on the developing nervous system can have deleterious effects that last a lifetime, long after exposure has ended, as the toxicant causes alterations of development of the nervous system [14].

Like many other insecticide compounds, pyrethroids are known to be neurotoxic [15]. In fact, intraperitoneal injection of 3-PBA in mice daily for 2 months has shown to induce synuclein aggregation in dopaminergic neurons, which may contribute to dopaminergic neurodegeneration [11]. Given its high lipophilicity, 3-PBA can cross the blood-brain barrier (BBB) and bioaccumulate in the brain, which is rich in lipids [11]. This increases the plausibility of its neurotoxic effects.

Additionally, a recent review provides relevant evidence which confirms that pyrethroids exposure during pregnancy may impact neurodevelopment for example by interference with thyroid hormone (TH) function [15].

Adverse effects on thyroid function warrant caution because THs play an important role in many aspects of human physiology including growth, development, energy metabolism, and reproduction [16,17]. Across vertebrates, particularly during pregnancy and the neonatal period, THs orchestrate metamorphosis, brain development, and metabolism. SPs and their metabolites have structural similarities with THs [18]. These similarities are believed to underlie SPs and 3-PBA interference with nuclear receptors of TH. In fact these compounds have been shown to inhibit TR-mediated gene expression [19]. A study evaluating environmental exposure to pyrethroids and thyroid hormones of pregnant women in Shandong, China, indicated that exposure to pyrethroids was widespread and negatively associated with serum concentrations of free triiodothyronine (FT3) [20]. Nevertheless, Zang et al., in a cohort of women in the first trimester of pregnancy, could not show an association between chemical exposure to pyrethroid pesticides during the early gestation period and maternal thyroid function [21]. There is a need to further explore the effects of pyrethroid exposure on thyroid function in pregnant women.

Some studies have evaluated urinary pyrethroid levels among pregnant women in different regions of the globe. In a randomized trial carried out in Idaho, USA, levels of 3-PBA were measured in 1^st^ trimester pregnant women who received either organic or conventional fruits and vegetables for consumption for 24 weeks. 3-PBA concentrations were significantly higher in urine samples collected from women in the conventional produce group compared to the organic produce group (0.95 vs 0.27 μg/L, *p* = 0.03) [22]. A study carried out in French pregnant women showed that among 5 pyrethroid metabolites, the urinary concentrations of 3-PBA were the highest with a mean concentration of 0.36 μg/L (0.50 μg/g creatinine) [23]. In China, a study carried out in pregnant women living in a rural area of the Jiangsu Province showed that median urinary 3-PBA concentration was 1.01 g/L (1.55 μg/g creatinine) [24].

A biomonitoring study conducted in the US has indicated that in recent decades, there has been an increase in pyrethroid insecticides home usage and a decrease in the use of organophosphorus pesticides (OP), resulting in detectable amounts of pyrethroid metabolites in population samples [25].

Human fertility rates are known to be decreasing both in developed and developing countries [26]. This reduction has been associated with socioeconomic changes and adverse lifestyle factors [27]. However, pesticide environmental contaminants have attracted international attention and recently came to be considered as possible contributors to human infertility [28].

Environmental exposure to pyrethroids can also adversely impact on pregnancy outcomes and offspring health, including newborn anthropometry, neurodevelopment, and behavioral problems [18]. A study of exposure to pyrethroid sprays during pregnancy has shown associations of this exposure with autism spectrum disorders (ASD) and developmental delay [29]. Cross-sectional studies also implicate pyrethroids in ASD [30] and Attention Deficit Hyperactivity Disorder (ADHD) [31].

Several previous studies of pyrethroid biomarkers and behavior have reported associations between pyrethroid levels and adverse behavioral problems in children. Although detection frequencies of pyrethroid metabolites were low, suggestive evidence that prenatal exposure to 3-PBA may be associated with a variety of behavioral and executive functioning deficits was found [32].

However, to date, there have been no studies evaluating the levels of exposure of Portuguese pregnant women to this pesticide neither its impact on maternal nor neonatal outcomes. So, the aims of this study were to characterize the exposure of pregnant women living in Portugal to 3-PBA and to evaluate the association of this exposure with maternal outcomes and newborn anthropometric measures. Additionally, considering that the exposure of the global population to 3-PBA is expected to vary with the seasons [24,33,34,35], with the maximum likelihood of exposure in summer [36], we decided to compare samples collected in summer with those collected in winter.

## 2. Materials and Methods

### 2.1. Ethical Approval

This study was performed according to the protocol approved by the Ethics Committee of São João University Hospital Center (CHUSJoão)/Faculty of Medicine of the University of Porto. Informed written consent was obtained from all study participants.

### 2.2. Study Design and Participants

**Figure 1:**
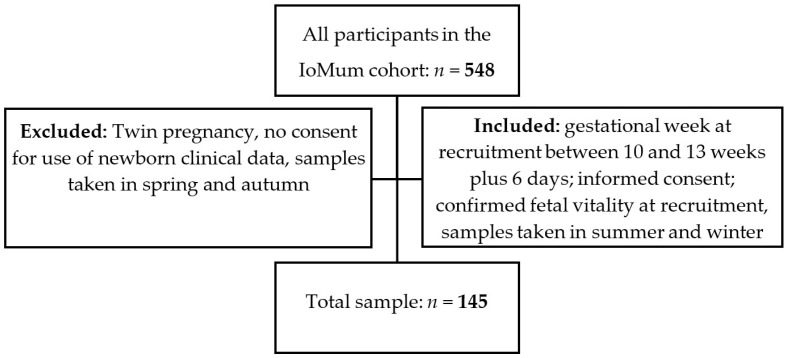
Recruitment and inclusion flowchart of the study.

A prospective observational study was carried out from the IoMum cohort (Monitoring iodine status in Portuguese pregnant women and the impact of supplementation—trial registration number NCT04010708) according to the guidelines laid down in the Declaration of Helsinki. Pregnant women attending their first trimester routine ultrasound scan at Centro Hospitalar Universitário de São João (CHUSJ), Porto, between April 2018 and April 2019 were invited to participate as described previously [37,38,39]. All women who had a routine ultrasound scan between 10 and 13 weeks of gestation with confirmed fetal vitality, who signed the informed consent form, and who provided a urine sample at recruitment in summer or in the winter were included in the study. Exclusion criteria were twin pregnancy, declaration of informed consent for use of the data of the newborn not being signed by the mother, and urine sample collection in spring or autumn (Figure 1).

Gestational age was determined from the measurement of the fetal crown-rump length.

At the time of enrollment between 10 and 13 weeks plus 6 days (timepoint 1, T1) and after informed consent, information was collected on various demographic and lifestyle factors, including age, area of residence, education, weight, and height of the pregnant, gestational age, smoking habits, and use of medicines. In the lifestyle questionnaire, food frequency information was obtained from a semi-quantitative food intake assessment questionnaire, where we verified the frequency of consumption of cow’s milk, yogurt, cheese, eggs, and fish.

At this time point (T1), a spot urine sample was also collected, and women were invited to a second contact with the IoMum team from 35 weeks until the end of gestation (time point 2, T2) for additional demographic and lifestyle information collection, spot urine collection and a finger prick blood spot. The urine samples were refrigerated upon collection and transported to the laboratory within the following 24 h for aliquot creation and freezing at −80 °C for future analysis.

Information collected at T2 falls outside the scope of this work, and so, it will not be further detailed. Information regarding both maternal and the newborn’s clinical details were obtained from the clinical records, including pregnancy outcomes and complications, mode of delivery, gestational age at delivery, and newborn’s anthropometric and vitality parameters.

### 2.3. Biochemical Analysis

#### 2.3.1. Chemical Elements Quantification

The analysis of 3-PBA was performed by gas chromatography with mass spectrometry (GC/MS), as described [2] 1 μL of sample was injected onto a Thermo Trace-Ultra gas chromatography, coupled to an ion trap mass detector Thermo Polaris, operated in the electron impact ionization at 70 eV. The ion source and the MS transfer temperature were set at 250 °C. Operating in the splitless mode (0.5 min), the helium was used as carrier gas at a constant flow rate of 1.3 mL min^−1^. The temperature of the injector was 240 °C. The column was a 30 m ZB-5MSi (0.25 mm i.d., 0.25 μm film thickness Zebron-Phenomenex), and oven temperature was programmed as described [2]. The analysis was developed in the SIM mode, based on the detection of selected ions for 3-PBA (141, 196, and 364).

Sample preparation was performed by solid-phase extraction (SPE). Briefly, the urine samples were thawed at room temperature. Then, a solution of urine in 1.5 mL of deionized water (H_2_O), 150 μL of sodium hydroxide (NaOH) (Merck), and 100 μL of the internal standard 2-phenoxy benzoic acid (2-PBA) (Sigma Aldrich) (1.5 mL + 1.5 mL + 150 μL + 100 μL, respectively) of were incubated at 37 °C for 15 min. After chemical deconjugation, the samples were transferred to the preconditioned SPE columns (Strata-X) (Phenomenex) with 5 mL methanol (MeOH) (Riedel de Haen) and 5 mL ammonium acetate (Merck). The columns were then immediately washed with 5 mL (MeOH): (H_2_O) (30/70 V/V). Following a short vacuum pulse to remove excess wash solution, the columns were dried under vacuum for 40 min using the SPE vacuum manifold. Elution was carried out with 5 mL of acidified MeOH (2% formic acid) (Carlo Erba), directly into a glass vial. Subsequently, the eluates were concentrated to 50 μL under a gentle stream of nitrogen.

3-PBA derivatization procedure was necessary prior to GC/MS analysis. The derivatization was performed by addition of 30 μL hexafluoro-2-propanol (HFIP) (Sigma Aldrich Darmstadt, Germany), 20 μL (N, N′-Diisopropylcarbodiimide (DIC) (Sigma Aldrich Darmstadt, Germany) and 400 μL of n-hexane (Merck, Darmstadt, Germany) to the 50 μL of the eluate obtained from the SPE extraction and vortex at room temperature during 10 min. In the final step, liquid-liquid extraction was performed with 1 mL of a 5% aqueous potassium carbonate solution (Panreac, Darmstadt, Germany) (to neutralize the excess derivatizing agent), shaken 5 min in the vortex, and finally, the supernatant was removed and placed in a vial with insert for injecting into GC/MS. The calibration curves and linear ranges of the detector response for 3-PBA were evaluated by analyzing the working standard solutions (15–200 μg L^−1^, 8 concentrations) in triplicate. In this study, the linearity, selectivity, the limit of detection (LOD) and limit of quantification (LOQ) were evaluated and the determinations that were below the LOD have been replaced by the constant LOD/2, according to Richardson, and Ciampi and Schisterman et al. [40,41,42]. LOD (0.364 μg/L) and LOQ (1.212 μg/L) were calculated as the minimum amount of analyte detectable with a signal-to-noise ratio (S/N) of 3 and 10, respectively; the linearity of the method was established by setting calibration curves using linear regression analysis over the concentration range. Selectivity was verified by comparing the chromatograms of the standards dissolved in n-hexane, the standards extracted from the spiked urine and the matrix blanks.

#### 2.3.2. Creatinine Quantification

Urine-based biomarkers are useful for assessing individuals’ exposure to environmental factors. However, inter-individual variations in urine concentration (which can be assessed by urinary creatinine) can directly affect urinary levels of contaminants. So, urinary creatinine was used to adjust 3-PBA urinary levels to urine concentration [43].

Measurements were performed using an ADVIA 1800 instrument according to the manufacturer’s instructions, based on the enzymatic reaction described by Fossati, Prencipe, and Berti [44]. 

Briefly, urinary creatinine was quantified by enzymatic conversion (creatininase) to creatine, which was then hydrolyzed by creatinase to produce sarcosine, and this decomposed by the sarcosine oxidase to form glycine, formaldehyde and hydrogen peroxide. The hydrogen peroxide formed produces a blue pigment through the action of peroxidase and by quantitative oxidative condensation with N-(3-sulfopropyl)-3-methoxy-5-methylaniline (HMMPS) and 4-aminoantipyrine. The creatinine concentration was obtained by measuring the absorbance of the blue color at 596/694 nm.

### 2.4. Maternal Outcomes and Newborn Anthropometric Measures

Maternal outcomes considered for association analyses with levels of 3-PBA were: medication for thyroid disease (as a proxy for hypothyroidism), glycemia in the first trimester, and type of delivery (women who had cesarean delivery, women who had a vaginal delivery).

For the categorization of variables of weight, head circumference and length of the newborn, percentile classification was used [45]. As a result, the newborns were classified into 3 categories regarding weight, head circumference, and length at birth:

- SGA: small for gestational age (below 10th percentile)
- AGA: appropriate for gestational age (between 10th percentile and 90th percentile)
- LGA: large for gestational age (above the 90th percentile)

### 2.5. Statistical Analysis

Descriptive statistics are presented as absolute and relative frequencies for categorical variables, mean and standard deviation (SD), or median and interquartile range (25th percentile (*P25*); 75th percentile (*P75*)) for continuous variables, depending on the symmetry of their distribution.

When testing hypotheses about continuous variables we used non-parametric tests (Mann-Whitney and Kruskal-Wallis tests) considering the hypotheses of non-normality and number of groups. When testing hypotheses on categorical variables, the chi-square test and the Fisher’s exact test were used, as appropriate.

The level of statistical significance was set at 5%, so the differences were considered statistically significant whenever *p* < 0.05. Statistical analyses were performed using SPSS^®^ v.28.0 (Statistical Package for the Social Sciences: Armonk, NY, USA).

## 3. Results

### 3.1. Sociodemographic Data

The sociodemographic characteristics of the study population are presented in Table 1. Most of the population resides in the municipalities corresponding to the coverage areas of CHUSJ (Maia (30%), Valongo (26%), and Porto (18%)), and a minority resides in other municipalities of the North of Portugal, including Matosinhos (*n* = 5 (4%), Vila Nova de Gaia (*n* = 4 (3%), and Vila do Conde (*n* = 3 (2%)). Forty five percent of the population has a low level of education (≤12 years), while around 19% of the population has higher degrees of education equivalent to a Master or PhD degree. The median age of the participants was 31.7 years old, with the youngest and the oldest participants being 19 and 40 years old, respectively. The median pre-pregnancy body mass index (BMI) was 23.6 kg/m^2^, and 65% of women were within the normal weight range (18.5–24.9 kg/m^2^). Calculation of BMI was based on the self-reported weights and heights of pregnant women 6 months before the day of recruitment. Regarding the number of pregnancies, 52% of women were primiparous. Only 7% of the pregnancies resulted in preterm births, and in total, this sample gave birth to 68 boys and 73 girls.

The mean (SD) weight at birth was 3152 (478) g, and 86% of newborns had adequate weights for their gestational ages. The median (*P25*; *P75*) urinary 3-PBA concentration was 0.182 (0.182; 0.372) µg/L, which is above the detection limit (LOD, 0.364 µg/L). To account for the urine concentration, we adjusted 3-PBA urinary excretion for creatinine concentration (median (*P25*; *P72*) of 0.263 (0.167; 0.458) µg/g and used this variable in all the remaining analyses.

Table 2 explores the association between urinary concentration of the pyrethroid metabolite 3-PBA with sociodemographic characteristics. The median concentration was the lowest in Porto, although the differences observed were not statistically significant. Maternal education level or smoking habits did not appear to be consistently associated with 3-PBA status. Regarding the BMI categories, a statistically significant difference was observed (*p* = 0.049), where the lowest medians were found in mothers with obesity. 

### 3.2. 3-PBA Exposure by Seasons

**Figure 2:**
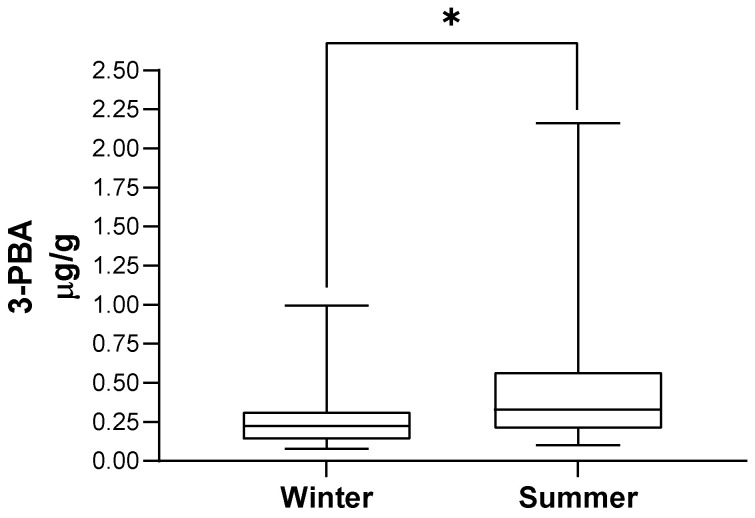
3-PBA urinary excretion according to season of urine collection. In each box, the central horizontal line marks the median value. * *p* < 0.001.

Table 3 presents the distribution of the population by season of urine collection and the corresponding 3-PBA urinary excretion, illustrated in Figure 2. 3-PBA with or without the adjustment for creatinine levels was higher in summer-collected urine (*p* < 0.001). In addition, we observed that 3-PBA detection rate was much higher in summer when compared with winter samples (53% (*n* = 39) versus 4% (*n* = 3); *p* < 0.001).

### 3.3. 3-PBA Exposure in Association with Food Intake

Table 4 shows the variation of urinary levels of 3-PBA, separated by seasons (summer and winter) with consumption of fish or yogurt. 3-PBA levels were found to be positively associated with the frequency of consumption of these foods in winter but not in summer. The frequency of consumption of other foods, such as cow’s milk, eggs, and cheese was not associated with 3-PBA levels (data not shown).

### 3.4. 3-PBA Levels and Maternal Outcomes

The association between 3-PBA levels and maternal and pregnancy clinical characteristics was studied.

Considering the structural similarities found between pyrethroids and THs and the consequent suspicion that they could antagonize thyroid hormone activities [19,21,46], levels of 3-PBA in our sample were analyzed according to medication for thyroid disease (as a proxy for hypothyroidism). We found that women who reported taking levothyroxine had higher median (*P25*; *P75*) urinary 3-PBA levels when compared to women who reported not having thyroid disease (0.534 (0.333; 0.976) μg/g, *n* = 9, and 0.266 (0.168; 0.430) μg/g, *n* = 124, respectively, *p* = 0.010).

Additionally, 1st trimester 3-PBA levels had a weak positive correlation with maternal fasting glycemia in the first trimester (*r =* 0.256; *p* = 0.011, Spearmen correlation), and women who had cesarean delivery had higher median (*P25*; *P75*) first trimester 3-PBA levels when compared with women who had vaginal delivery (0.302 (0.206; 0.528) μg/g versus 0.255 (0.153; 0.410) μg/g, respectively, *p* = 0.041).

### 3.5. Neonatal Outcomes

Median 3-PBA concentrations were very similar between male and female offspring (Table 5).

Table 6 shows the variation of urinary 3-PBA levels with neonatal outcomes; the described pyrethroid pesticide metabolite was not associated with anthropometric categories at birth.

## 4. Discussion

Analysis of results for 3-PBA urinary excretion in pregnant women showed concentrations above LOD in 29% of the sample population.

3-PBA urinary excretion tended to associate with residence area, being lower in Porto, when compared to other municipalities. The fact that Porto is a predominantly urban city when compared with Valongo [47], for example, which has a great amount of rural territory, could account for this trend. In line with this rationale, Wielgomas et al. found that the detection of metabolites of SPs in preschool and school children and their parents was more frequent and with higher urinary excretion in rural when compared with urban areas in Poland [48]. In that study, 3-PBA was detected in 77% and 94% of samples from urban and rural areas, respectively, and curiously, 3-PBA urinary levels found in rural and urban areas were very similar to those found in our study (0.272 vs 0.155 µg/g for rural vs urban areas in Poland [48], respectively; 0.278 vs 0.174 µg/g for Porto vs Valongo in Portugal, respectively). Concerning frequency of exposure to 3-PBA, our study revealed a low detection rate (29%) when compared, for example, with reports for Polish, American, French, or Chinese population samples [22,23,24,48]. On the other hand, the 3-PBA detection rate herein described is comparable or higher than those obtained in studies from Germany [49,50,51], Spain [52,53], or France [54,55]. The variation in concentration 3-PBA medians, LOD/LOQ values or rates of detection across studies can be attributable to local exposure characteristics but, importantly, to heterogeneity in urine sampling, sample preparation, quantification methods, and reporting [56]. Altogether, these findings corroborate the claim recently published by Andersen et al. [56] for the need for guidelines to harmonize quantification methods and reporting in human biomonitoring studies.

Regarding pre-gestational BMI categories, 3-PBA levels were the lowest in mothers with obesity. Being highly lipophilic, 3-PBA conjugates with lipids such as cholesterol, bile acids, and triglycerides, which results in 3-PBA retention in organs particularly rich in lipid content [57] such as adipose tissue. This retention could result in the observed decrease in 3-PBA urinary excretion with increasing BMI (and fat mass).

Yoo M et al. also reported a negative association between 3-PBA levels and BMI for high levels of exposure in Korean adults [58]. Despite this, other studies have shown either a positive or no association between 3-PBA urinary excretion and BMI in pregnant women [59] or in the elder, respectively. We cannot currently explain the disparity of these results when compared to ours.

Importantly, 3-PBA exposure was found to be higher in the summer, when compared to winter-collected urine samples. Several studies have shown that urinary pyrethroid metabolite levels of pregnant women follow trends of seasonal insecticide use related to pest management practice [24,33,34,35,36]. A study carried out in China also observed a trend of seasonal variation, with levels of urinary metabolites in the summer significantly higher than those in the winter. These data indicate the need to assess the potential adverse effects of exposure to pyrethroid pesticides on fetuses and infants, in order to take appropriate measures to protect pregnant women from higher exposure to pesticides in more susceptible seasons [24]. Despite this, we cannot exclude that seasonal variations in 3-PBA levels could also be due to seasonal variations in fruits or vegetables consumption. In fact, a systematic review with metanalysis has shown that fruit consumption across the world tends to be higher in autumn and winter, but vegetable consumption tends to be higher in spring and summer [60]. This may, in all probability, result in differential SPs exposure according to seasons.

In relation to food consumption, 3-PBA levels were associated with a higher consumption of fish and yogurt in winter-collected samples; the lack of association between food consumption and 3-PBA urinary levels in summer-collected samples could be due to a greater exposure to environmental 3-PBA in the summer, which could mask the foodborne exposure.

Although the available evidence regarding associations between dairy or fish consumption and 3-PBA urinary levels is currently weak, 3-PBA-parent pesticides, such as cypermethrin, bifenthrin, and cyhalothrin have been detected both in fish and in dairy samples [61,62] and our data show that these foods could act as vehicles for 3-PBA exposure. In fact, 3-PBA levels were 4.5 times higher in the group of people consuming fish more than 3 times a week and 1.5 times higher in the group of people consuming at least one yogurt a day. Despite the magnitude of the difference and the statistical significance, association of 3-PBA levels with fish consumption should be interpreted with caution, because of the small sample size in the category of fish consumption >3 times/week.

In addition, we cannot explain the reason why yogurt was the only dairy food which consumption associated with 3-PBA levels. Despite this, our data suggest that fish and yogurt may represent a form of pyrethroid bioaccumulation.

With respect to the association of 3-PBA with maternal clinical characteristics, we observed higher levels of 3-PBA in women medicated for hypothyroidism, which corroborates the idea that pyrethroids, whose structure is similar to THs, could associate with thyroid dysfunction, as suggested by others [19,46]. However, in our study, only 9 women are included in the hypothyroidism medicated group, and so, these results should be read with caution.

It is estimated that at least 10% of approved pesticides in the European Union (EU) possess endocrine disruption (ED) properties [63,64]. Experimental studies have reported that many currently used pesticides (or their metabolites) may interfere with the hypothalamic-pituitary-gonadal and hypothalamic-pituitary-thyroid axes [46,65]. Especially among vulnerable human populations, such as developing fetuses and infants, changes in THs beyond the reference range may cause a significant adverse impact on health, including the development of neurodevelopmental problems [16,66]. However, the effects of current human pyrethroid exposure levels on reproductive and thyroid function are poorly understood.

Still, with regard to maternal clinical characteristics, there was a weak but statistically significant correlation between 3-PBA levels and maternal fasting glycemia in the first trimester. This result is in line with a study conducted in China that studied the association between serum levels of pyrethroid insecticides and risk of type 2 diabetes which found that high concentrations of serum pyrethroid insecticides were significantly associated with an increased risk of type 2 diabetes [67]. A US study suggests that exposure to pyrethroids, as estimated by urinary 3-PBA concentrations, was associated with an increased risk of diabetes in the general adult population [68]. Hansen et al. found a severely increased prevalence of prediabetes among Bolivian pesticide sprayers compared with a control group. Within the sprayer group, an association between cumulative exposure to pyrethroids and abnormal glucose regulation was seen [69].

Finally, in our study, we found no associations between pyrethroid pesticide metabolite levels and the anthropometric profile of newborns at birth. Like us, Berkowitz et al., found no significant association between newborn anthropometric measures (birth weight, length, and head circumference) and maternal 3-PBA urinary in early pregnancy [70]. In a prospective birth cohort in rural northern China between September 2010 and 2012, no associations were found between 3-PBA levels and birth length, head circumference, or gestational duration [18]. Contrarily, a study conducted in New York City aimed to investigate the association between delivery outcomes and urinary biomarkers of pyrethroids among healthy pregnant women and found that 3-PBA concentrations were positively associated with head circumference in boys (*p* = 0.53, 95% CI: 0.03, 1.04) [3]. Also, in a study carried out in northern China, aimed at linking pesticides and other environmental exposures with the health of pregnant women and their children, it was observed that the total levels of pyrethroid metabolites in the mother’s urine maternal urine were positively associated with birth weight and head circumference [71].

One main limitation of this study was the small sample size, which resulted from the selection of samples collected during the summer and winter and exclusion of spring- or autumn-collected samples. In addition, the initial questionnaire did not ask pregnant women about the frequency of consumption of other foods, such as fruits and vegetables, and exposure to additional agents that may contain environmental pollutants.

Another limitation was that we could not analyze occupational exposure to 3-PBA due to lack of detailed information regarding the profession of the participants. In fact, information regarding professional occupations was extracted from clinical registries with no possible association with the likelihood of exposure.

A strong point of our study was that, although the recruitment was carried out at central hospital, we could invite pregnant women undergoing routine prenatal surveillance, not being restricted to pregnant women in hospital consultation, and therefore, we included women with and without pathology.

## 5. Conclusions

In conclusion, the present study characterized 3-PBA status in a sample of pregnant women living in the Porto metropolitan area. Nonetheless, 3-PBA excretion associated negatively with maternal pre-pregnancy BMI, suggesting 3-PBA adipose tissue retention. Our data also suggest that 3-PBA exposure is higher during the summer, and food such as fish and yogurt may be a source of 3-PBA dietary exposure, particularly when environmental exposure is low.

As to clinical features, our data suggest that 3-PBA may be associated with thyroid dysfunction, which could be due to a thyroid hormone antagonistic effect, as previously described by other authors. This conclusion should be considered cautiously given the small sample size of the hypothyroidism medicated group in our study.

In fact, this data, together with the association herein found between 3-PBA and fasting glycemia, deserve future attention.

Finally, in this work, we did not find an association between 3-PBA maternal urinary excretion in the first trimester and anthropometric measures of the newborn.

This study highlights that pregnant women living in Portugal may be exposed to 3-PBA and that this exposure may associate with maternal clinical features during pregnancy. More studies are needed to confirm data regarding association of 3-PBA exposure and newborn outcomes and to analyze the impact of these exposures on long-term maternal or childhood outcomes.

## Figures and Tables

**Table 1:** General characteristics of the study sample (*n* = 145).

**Residence Area**, *n* (%)		
Maia	41	(30)
Porto	25	(18)
Valongo	35	(26)
Outros	35	(26)
**Maternal education level**, *n* (%)		
Low (≤12 years)	62	(45)
Medium (13–15 years)	50	(36)
High (≥16 years)	26	(19)
**Age (years)**, *n*	145	
Mean	32	
SD	5.2	
**Pre-pregnancy BMI (kg/m^2^)**, *n*	142	
Median	24	
*P25*; *P75*	21; 26	
Minimum	16	
Maximum	36	
**Pre-pregnancy BMI categories**, *n* (%)		
Low weight	11	(8)
Normal weight	92	(65)
Overweight	24	(17)
Obesity	15	(10)
**Gestational age at recruitment (weeks)**, *n*	145	
Median	12	
*P25*; *P75*	12; 13	
**Primiparous**, *n* (%)		
No	70	(48)
Yes	75	(52)
**Preterm (37 weeks)**, *n* (%)		
No	131	(93)
Yes	10	(7)
**Newborn sex**, *n* (%)		
Male	68	(48)
Female	73	(52)
**Birth weight (grams)**, *n*	141	
Mean	3152	
SD	477	
**Birth weight classification**, *n* (%)		
SGA	12	(9)
AGA	122	(86)
LGA	7	(5)
**3-PBA (µg/L)**, *n*	145	
Median	0.182	
*P25*; *P75*	0.182; 0.372	
<LOD, *n* (%)	103	(71)
≥LOD, *n* (%)	42	(29)
**3-PBA (µg/g)**, *n*	145	
Median	0.263	
*P25*; *P75*	0.167; 0.458	

SGA, small for gestational age; AGA, appropriate for gestational age; LGA, large for gestational age. LOD, limit of detection. Missing’s: between 2 and 6%.

**Table 2:** Urinary levels of 3-PBA (µg/g) by participant characteristics.

	*n*	(%)	*P25*	Median	*P75*	*p*
**Residence Area**						
Maia	41	(30)	0.174	0.274	0.450	0.056 ^a^
Porto	25	(18)	0.131	0.174	0.294	
Valongo	35	(26)	0.172	0.278	0.368	
Other	35	(26)	0.172	0.333	0.507	
**Maternal education level**						
Low (≤12 years)	62	(45)	0.166	0.278	0.525	0.242 ^a^
Medium (13–15 years)	50	(36)	0.172	0.310	0.410	
High (≥16 years)	26	(19)	0.139	0.189	0.368	
**Smoking habits**						
Non-Smoker	94	(66)	0.172	0.276	0.497	0.185 ^a^
Smoker	23	(16)	0.169	0.254	0.309	
Former smoker	26	(18)	0.142	0.206	0.334	
**Pre-pregnancy BMI categories**						
Underweight	11	(8)	0.152	0.291	0.328	0.049 ^a^
Normal weight	92	(65)	0.188	0.293	0.507	
Overweight	24	(17)	0.165	0.241	0.340	
Obesity	15	(11)	0.103	0.155	0.346	

Classification of the residence Area based on Instituto Nacional de Estatística de Portugal I.P., 2014. ^a^ Kruskal-Wallis. Missing’s: between 2 and 6%.

**Table 3:** Levels of creatinine and 3-PBA with and without adjustment of creatinine in winter and summer samples.

			Creatinine (mg/dL)				3-PBA (µg/L)					3-PBA (µg/g)				
Seasons	*n*	(%)	*P25*	Median	*P75*	*p*	*P25*	Median	*P75*	(Min; Max)	*p*	*P25*	Median	*P75*	(Min; Max)	*p*
Summer	74	51	46.06	79.61	121.81	0.397 ^a^	0.182	0.371	0.394	(0.182; 0.553)	<0.001 ^a^	0.209	0.331	0.556	(0.082; 2.166)	<0.001 ^a^
Winter	71	49	58.47	85.35	133.75		0.182	0.182	0.182	(0.182; 0.390)		0.139	0.218	0.311	(0.780; 1.064)	

^a^ Mann-Whitney.

**Table 4:** 3-PBA levels in association with food intake.

Food Intake	3-PBA Summer (µg/g)					3-PBA Winter (µg/g)				
	*n*	(%)	Median	*(P25; P75)*	*p*	*n*	(%)	Median	*(P25; P75)*	*p*
**Fish**										
≤3 times a week	41	(32)	0.318	(0.228; 0.608)	0.533 ^a^	67	(52)	0.213	(0.139; 0.301)	0.002 ^a^
>3 times a week	17	(13)	0.325	(0.155; 0.650)		3	(2)	0.976	(0.343; 1.064)	
**Yogurt**										
≤6 times a week	40	(28)	0.350	(0.236; 0.521)	0.938 ^a^	40	(28)	0.180	(0.133; 0.270)	0.015 ^a^
≥1 time a day	33	(23)	0.328	(0.174; 0.618)		30	(21)	0.269	(0.170; 0.462)	

**^a^** Mann-Whitney. Missing’s: between 1 and 23%.

**Table 5:** 3-PBA levels and newborn sex.

Newborn Sex	*n*	(%)	*P25*	Median	*P75*	(Min; Max) µg/g	*p*
Male	68	(48)	0.161	0.271	0.401	(0.078; 1.428)	0.463 ^a^
Female	73	(52)	0.170	0.270	0.507	(0.780; 2.166)	

**^a^** Mann-Whitney. Missing’s: 3%.

**Table 6:** Maternal first trimester urinary 3-PBA levels and newborn outcomes.

Birth Size Categories	*n*	(%)	*P25*	Median	*P75*	*p*
**Birth weight**						
SGA	12	(8)	0.196	0.303	0.356	
AGA	122	(87)	0.167	0.261	0.462	0.786 ^a^
LGA	7	(5)	0.174	0.301	0.497	
**Birth head circumference**						
SGA	13	(10)	0.203	0.278	0.333	0.973 ^a^
AGA	112	(83)	0.166	0.263	0.463	
LGA	10	(7)	0.168	0.257	0.456	
**Birth length**						
SGA	9	(6)	0.221	0.263	0.291	0.171 ^a^
AGA	131	(93)	0.168	0.274	0.476	
LGA	1	(1)	0.101	0.101	0.101	

SGA, small for gestational age; AGA, adequate for gestational age; LGA, large for gestational age. **^a^** Kruskal-Wallis. Missing’s: between 3 and 7%.

## Data Availability

The data sets generated during and/or analyzed during the current study are available from the corresponding author on reasonable request.
